# 1378. Seroprevalence and risk factors for cysticercosis in Mexican-Americans in Starr County, Texas

**DOI:** 10.1093/ofid/ofad500.1215

**Published:** 2023-11-27

**Authors:** Megan M Duffey, Elise M O’Connell, Lauren M Leining, Nina L Tang, Craig L Hanis, Eric L Brown, Sarah M Gunter

**Affiliations:** Baylor College of Medicine, Houston, Texas; National Institute of Allergy and Infectious Diseases, Bethesda, Maryland; University of Texas School of Public Health, Austin, Texas; National Institute of Allergy and Infectious Diseases, Bethesda, Maryland; University of Texas School of Public Health, Austin, Texas; University of Texas School of Public Health, Austin, Texas; Baylor College of Medicine, Houston, Texas

## Abstract

**Background:**

Neurocysticercosis (NCC) is a parasitic infection caused by the tapeworm *Taenia solium*, and is a leading cause of epilepsy and chronic headaches in endemic areas including Latin America, Africa, and Asia. The epidemiology of NCC has not been well-studied in the United States to date, but small epidemiological studies suggest evidence of this disease in populations living in poverty and who have immigrated from an endemic region. There is a population living in Starr County, Texas along the Texas-Mexico border in whom the presence of other neglected tropical diseases has been demonstrated due to the intersection of poverty, climate, and high rate of immigration.

**Methods:**

Our study was a serologic survey and risk factor analysis of cysticercosis in a pre-existing cohort of Mexican-Americans in Starr County, Texas. We used a triplex enzyme-linked immunoassay (ELISA) against cysticercosis-specific antigens and self-reported survey data and neighborhood-level variables to conduct a risk-factor analysis of seropositive participants.

**Results:**

We identified an overall seropositivity to *T. solium* in 7.4% (45/605) of the cohort which likely represents a combination of parasite exposure, *T. solium* disease outside of the central nervous system, old calcified NCC, and viable or degenerating NCC. Female gender, specific occupation and indoor versus outdoor occupation were found to be significantly associated with cysticercosis seropositivity. Thirty-nine of the 45 seropositive participants were female (p 0.021), 26 were employed in healthcare, caregiving, or social service (p 0.009), and 42 were employed in an indoor occupation (p < 0.001). These occupations could pose a risk for *T. solium* transmission. A geospatial analysis of cases at the census tract level showed increased percentage of seropositivity centered around urban cities Rio Grande City and Roma, as well as smaller city La Grulla and census-designated place Alto Bonito.

**Conclusion:**

There is a critical need to investigate these cysticercosis-seropositive cases with brain imaging to evaluate for neurocysticercosis, as well as conduct an epidemiological investigation to determine disease transmission in Starr County.

**Disclosures:**

**All Authors**: No reported disclosures

